# Derivation of integration‐free iPSCs from a Klinefelter syndrome patient

**DOI:** 10.1007/s12522-015-0213-9

**Published:** 2015-07-03

**Authors:** T. Shimizu, M. Shiohara, T. Tai, K. Nagao, K. Nakajima, H. Kobayashi

**Affiliations:** ^1^ Department of Urology Toho University School of Medicine 6‐11‐1 Omori‐Nishi, Ota‐ku 143‐8541 Tokyo Japan

**Keywords:** Cardiomyocyte, iPS, Klinefelter syndrome, Stem cells, Testis

## Abstract

**Purpose:**

Klinefelter syndrome (KS) (47, XXY) is the most common sex chromosome abnormality in humans. KS is characterized by gynecomastia, tall stature, small testes, low testosterone levels, learning disabilities, and behavioral problems. KS is also associated with infertility due to non‐obstructive azoospermia (NOA). The mechanism underlying NOA is still poorly understood, and although there is no current treatment, the use of microdissection testicular sperm extraction (micro‐TESE) followed by in vitro fertilization can result in successful conception. The generation of induced pluripotent stem (iPS) cells derived from KS patients may be useful for studying the disease mechanism and identifying novel therapies.

**Methods:**

Cells from a KS patient were transduced with Sendai viral vectors encoding four transcription factors, OCT4, SOX2, KLF4, and C‐MYC, and the transduced cells were analyzed for in vitro and in vivo pluripotency.

**Results:**

KS patient‐derived iPS cells were successfully generated and shown to produce teratomas in the testes of SCID mice. In vitro differentiation of the iPS cells into cardiomyocyte‐like cells was confirmed by the presence of clusters of beating cells.

**Conclusions:**

KS patient‐derived iPS cells that could differentiate into cardiomyocyte‐like cells were established.

## Introduction

Klinefelter syndrome (KS), or 47, XXY syndrome, is the most common sex chromosome abnormality found in humans, occurring in approximately 1 in 500–1000 live deliveries [1]. KS is characterized by gynecomastia, tall stature, small testes, low testosterone levels, learning disabilities, and behavioral problems. Affected men suffer from KS‐associated progressive testicular failure (resulting in azoospermia or cryptozoospermia), small testes (5–7 cm^3^), and low testosterone levels. In addition, individuals with KS have an increased risk for various diseases, including diabetes, cardiovascular disease, and cancer, and their offspring have an increased risk for chromosomal abnormalities [1]. Thus, it is recommended that individuals with KS symptoms undergo prompt cytogenic evaluation.

The KS karyotype arises spontaneously when paired X chromosomes fail to separate during the meiosis stage of oogenesis or spermatogenesis [2]. In addition, a small percentage (<3 %) of X chromosome polysomy occurs during early divisions of the fertilized egg, while more than 10 % occurs as a result of postfertilization nondisjunction [3]. It is predicted that the aberrant expression of X chromosome‐linked genes plays a role in the spermatogenesis defect seen in KS patients. However, the mechanism underlying the infertility of KS is still poorly understood, and the only treatment for the infertility is microdissection testicular sperm extraction (micro‐TESE), which can result in successful in vitro fertilization.

The development of induced pluripotent stem (iPS) cells holds much promise for regenerative medicine. Two seminal studies showed that human somatic cells can be reprogrammed into pluripotent stem cells by introducing a set of transcription factors that regulate pluripotency [4, 5]. iPS cells possess a nearly unlimited capacity for self‐renewal and pluripotency, similar to embryo‐derived embryonic stem (ES) cells. In addition, iPS cells are associated with fewer ethical concerns than ES cells. The iPS cells derived from KS patients may be useful for studying the pathogenic mechanisms of the disease.

We previously obtained iPS cells derived from a KS patient using the lentiviral system [6]; however, the differentiation of these cells into neurons or cardiomyocytes was unsuccessful. In the current study, we used a Sendai virus vector, which expressed four transcription factors (OCT4, SOX2, KLF4, and C‐MYC), to generate iPS cells derived from the testicular tissue of a patient with KS. We confirmed the multipotency of the resulting iPS cells by demonstrating their ability to differentiate into cardiomyocyte‐like cells.

## Materials and methods

### Human subjects

Written informed consent was obtained from a KS patient who visited the Reproduction Center of Toho Medical Center, Omori Hospital, and this study was approved by the Ethics Committee of Toho University School of Medicine (24056‐18016 revision). Testicular tissue of the KS patient was obtained by micro‐TESE. We analyzed the tissue for the presence of sperm cells, but could not confirm their presence.

### Human KS testis tissue

KS testicular tissue was composed of the rete testis, Sertoli cells, Leydig cells, and fibroblasts. The fresh tissue was subjected to enzymatic digestion [7], and the dissociated cells were then plated on 10‐cm tissue culture dishes coated with 0.1 % gelatin (Sigma) in a standard culture medium [Dulbecco's modified Eagle's medium (DMEM, Invitrogen) containing 7 % fetal bovine serum (FBS), 2 mM glutamine (Sigma), and antibiotics (50 U/ml penicillin and 50 μg/ml streptomycin, Sigma)]. These cells were incubated at 37 °C in a humidified atmosphere containing 5 % CO_2_ for 14 days, resulting in the establishment of a KS testicular fibroblast line.

### Cell culture

Mitomycin C‐treated mouse embryonic fibroblast (MEF) feeder cells were purchased (ReproCELL) and plated in 0.1 % gelatin‐coated 10‐cm culture dishes in the standard culture medium. iPS cells were generated as described below and maintained in Primate ES medium (ReproCELL), supplemented with 4 ng/ml recombinant human basic fibroblast growth factor (bFGF) (R&D Systems). The iPS cells were grown in Primate ES cell medium supplemented with 4 ng/ml bFGF and 10 μM Y27632 (Wako), and the medium was changed every day. For passaging, the iPS cells were rinsed once with Hank's balanced salt solution (HBSS) (Invitrogen) and incubated in dissociation medium (ReproCELL) at 37 °C. All cultures were maintained at 37 °C in a humidified atmosphere containing 5 % CO_2_.

### iPS cell generation

To generate iPS cells, CytoTune^R^‐iPS 2.0, a kit containing four Sendai viruses (SeV), encoding OCT4, SOX2, KLF4, and C‐MYC (DNAVEC) was used. The KS testicular fibroblasts were incubated in SeV‐containing medium for 24 h, followed by incubation with standard medium, which was replaced every day. Seven days later, the cells were harvested by trypsinization, and 5 × 10^4^ cells were placed on MEF feeder cells in a 10‐cm dish in Primate ES cell medium supplemented with 4 ng/ml bFGF. The medium was changed every day, and the cells were monitored daily for morphological changes.

### Reverse‐transcription (RT)‐PCR

Total RNA was prepared using the PureLink Micro‐to Midi Total RNA Purification System (Invitrogen). RT‐PCR was performed using the SuperScript III One‐Step RT‐PCR System with Platinum^R^ Taq DNA Polymerase (Invitrogen) to analyze the expression of pluripotency markers. The following conditions were used: 55 °C for 30 min for reverse transcription, 94 °C for 2 min to inactivate the reverse transcriptase and activate the polymerase, 40 cycles at 94 °C for 15 s, 55 °C for 30 s, 68 °C for 1 min, and 68 °C for 5 min for the final extension. The following products were analyzed: *OCT4* (315 bp), *NANOG* (285 bp), and *GAPDH* (513 bp). The primers were as follows: *OCT4*, 5′‐GAA GGT ATT CAG CCA AAC GAC‐3′ (forward) and 5′‐GTT ACA GAA CCA CAC TCG GA‐3′ (reverse); *NANOG*, 5′‐TGC AAA TGT CTT CTG CTG AGA T‐3′ (forward) and 5′‐GTT CAG GAT GTT GGA GAG TTC‐3′ (reverse); *GAPDH*, 5′‐GTC CAT GCC ATC ACT GCC A‐3′ (forward) and 5′‐TTA CTC CTT GGA GGC CAT G‐3′ (reverse) [8, 9].

### Immunostaining and immunofluorescence microscopy

To analyze the expression of stem cell markers, the Human Embryonic Stem Cell Marker Antibody Panel was used (R&D Systems). The cells were washed twice with phosphate‐buffered saline (PBS), fixed with 4 % (w/v) paraformaldehyde for 20 min, permeabilized for 60 min with PBS containing 0.1 % (v/v) Triton X‐100, and then blocked for 3 h with PBS containing 20 % donkey serum. The fixed samples were incubated with anti‐human alkaline phosphatase monoclonal, anti‐human NANOG polyclonal, anti‐human OCT4 polyclonal, anti‐human SSEA‐1 monoclonal, and anti‐human SSEA‐4 monoclonal antibodies (all from R&D Systems) as indicated, and then washed three times with PBS containing 0.1 % (v/v) Triton X‐100, and probed with the appropriate secondary antibodies (Alexa 488‐conjugated anti‐goat IgG antibody or Alexa 488‐conjugated anti‐mouse IgG antibody) (Molecular Probes). Nucleic acids were stained with SYTOX^R^ orange nucleic acid stain (Molecular Probes).

### In vitro pluripotency assessment

Confluent iPS cells in a 6‐cm dish were harvested by trypsinization and transferred to Poly (hydroxyethyl methacrylate‐co‐methyl methacrylate; HEMA‐MMA)‐coated six‐well dishes in primate ES cell medium without bFGF. The medium was changed every other day, and the cells were maintained in floating culture for 8 days. The cells were then placed on 0.1 % gelatin‐coated six‐well dishes and incubated with ES medium for 8 days. After embryoid body formation, we confirmed the cells’ ability to differentiate in vitro by examining the expression of differentiation markers by immunocytochemistry. The cells were washed twice with PBS, fixed with 4 % (w/v) paraformaldehyde for 20 min, permeabilized for 60 min with PBS containing 0.1 % (v/v) Triton X‐100, and then blocked for 3 h with PBS containing 20 % donkey serum. Anti‐α‐fetoprotein mouse IgG (R&D Systems), anti‐α‐smooth muscle actin mouse IgG (Dako), and anti‐βIII‐tubulin mouse IgG (Chemicon) were used to analyze the expression of endodermal, mesodermal, and ectodermal markers, respectively. The cells were then washed three times with PBS containing 0.1 % (v/v) Triton X‐100, followed by incubation with an Alexa 488‐conjugated anti‐mouse IgG antibody (Molecular Probes). Nucleic acids were stained with SYTOX^R^ orange nucleic acid stain (Molecular Probes).

### In vivo pluripotency assessment

Approval was obtained from the Animal Committee of Toho University School of Medicine. The registration certificate number is “14‐53‐186.” Confluent iPS cells in a 6‐cm dish were harvested by trypsinization, collected in tubes and centrifuged, and the pellets were suspended in ES medium supplemented with 10 μm Y27632. The cells were then injected into the testes of 8‐week‐old SCID mice (Charles River). Three months after injection, the mice were examined for teratoma formation. The identified tumors were dissected and fixed with 10 % formalin. Pathological analyses were performed by the Tokyo Central Pathology Laboratory, Japan.

### Cardiomyocyte‐like cell differentiation of the KS patient‐derived iPS cells

To induce the KS iPS cells to differentiate into cardiomyocyte‐like cells, we used PSdif‐Cardio^R^ (StemRD). Briefly, KS iPS cells were cultured under feeder‐free conditions on thin layer Matrigel‐coated plates (BD Biosciences). The cells were incubated successively in PSdif‐Cardio^R^ A, B, and C media (according to the manufacturer's instructions) to induce cardiogenesis. Clusters of beating cells typically appeared approximately 14 days after induction.

## Results

### Generation of iPS cells derived from the testicular tissue of a patient with KS

**Figure 1:**
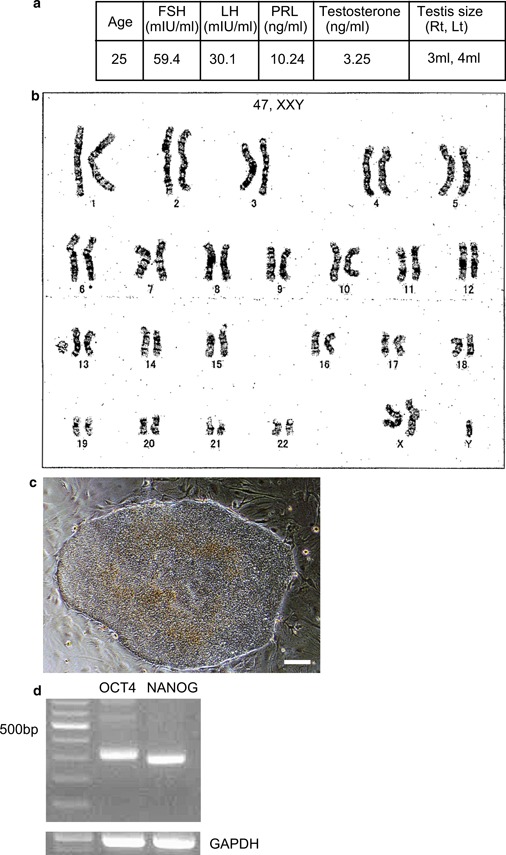
Generation of iPS cells derived from the testicular tissue of a KS patient. The isolated cells were transduced with an SeV construct expressing four transcription factors (OCT4, SOX2, KLF4, and C‐MYC). **a** Overview of the KS patient. **b** G‐banding chromosome analysis of the KS patient. **c** Morphology of the KS patient‐derived iPS cells. *Bars* = 60 μm. **d** RT‐PCR analysis of *OCT4*, *NANOG*, and *GAPDH* mRNA expression in the iPS cells. *GAPDH* mRNA was used as a loading control

Testicular tissue was obtained from a patient with KS by micro‐TESE (Fig. 1a). G banding of the patient's chromosomes indicated the presence of an extra X chromosome (47, XXY) (Fig. 1b). The tissue was processed and the isolated cells were cultured and subjected to iPS induction by transduction with a set of SeV constructs (see “Materials and methods”). Cells with an ES cell‐like morphology were first visible 15–16 days after transduction. The resulting colonies were maintained under human ES cell culture conditions (Fig. 1c). In this experiment, we obtained approximately 20 ES cell‐like colonies from 5 × 10^4^ cells (data not shown). The colonies were collected 21–25 days after transduction and transferred into a 24‐well plate with MEF feeder cells in Primate ES cell medium containing bFGF and Y27632. The morphology of the KS‐derived cells was similar to that of human ES cells. We confirmed that these colonies were 47, XXY by G‐banding (data not shown). From these colonies, which represented multiple sub‐cloned cell lines from the patient, we selected one cell line for further characterization.

### KS‐derived iPS cells express stem cell markers

**Figure 2:**
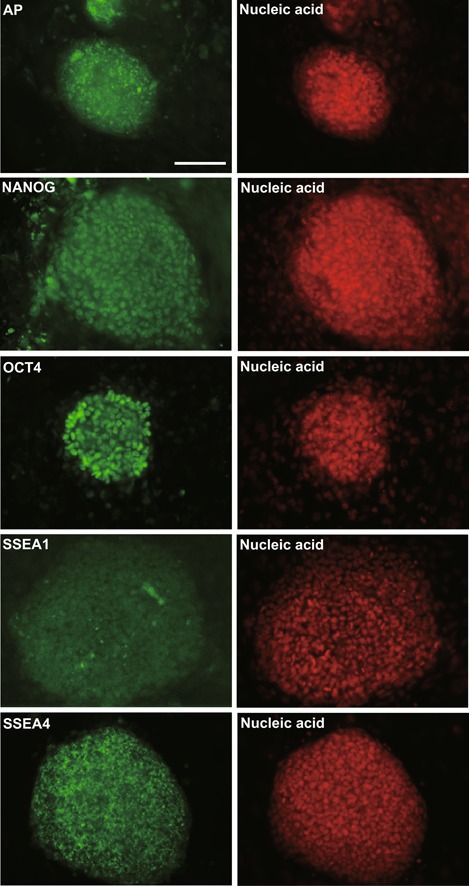
Immunostaining analysis of the KS patient‐derived iPS cells. Nuclei were stained with SYTOX^R^ Orange. *Bars* = 100 μm

We confirmed that the ES‐like cells derived from the KS patient expressed the undifferentiated ES cell markers *OCT4* and *NANOG* (Fig. 1d). In addition, we showed that the ES‐like cells did not express the exogenous transgenes (*OCT4*, *SOX2*, *KLF4*, and *C*‐*MYC*) or genes encoded by the SeV genome (data not shown). We also performed immunocytochemistry to examine the protein expression of ES cell markers in the iPS cells. We showed that these cells expressed alkaline phosphatase (AP), NANOG, OCT4, and SSEA‐4, but not SSEA‐1 (Fig. 2).

### KS‐derived iPS cells are multipotent in vitro

**Figure 3:**
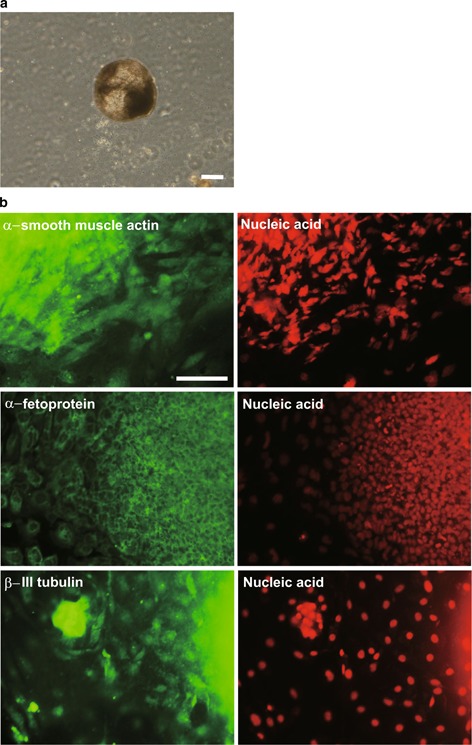
Embryoid body formation and in vitro differentiation of the KS‐derived iPS cells. **a** Embryoid bodies derived from the iPS cells in vitro. *Bars* = 30 μm. **b** Immunostaining analysis of the embryoid bodies and depiction of the three germ layers. Nuclei were stained with SYTOX^R^ Orange. *Bars* = 100 μm

To investigate the multipotency of the KS‐derived iPS cells, we examined their ability to form embryoid bodies in vitro. After culturing the cells for 8 days under floating culture conditions, the presence of embryoid bodies was confirmed (Fig. 3a). To examine the expression of markers for the three germ layers, we analyzed the plate‐attached embryoid bodies by immunocytochemistry. We confirmed that the embryoid bodies expressed α‐smooth muscle actin, α‐fetoprotein, and β III‐tubulin (Fig. 3b).

### KS‐derived iPS cells are multipotent in vivo

**Figure 4:**
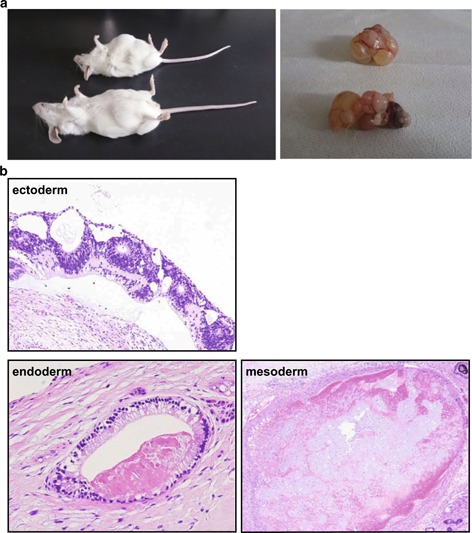
Teratoma formation after transplantation of the KS‐derived iPS cells into the testes of SCID mice. **a** Teratoma formation. **b** Hematoxylin and eosin staining of sections of the KS‐derived teratomas

To examine the differentiation potential of the iPS cells in vivo, we transplanted the cells into the testes of SCID mice. We monitored the formation of tumors in the testes for 3 months after the injection and confirmed the presence of tumors during this time period (Fig. 4a). Histological analysis of the tumors showed that the KS‐derived iPS cells were capable of differentiating into the three germ layers in vivo (Fig. 4b).

### Cardiomyocyte‐like cell differentiation of iPS cells derived from KS

To examine the ability of the KS‐derived iPS cells to differentiate into structurally and functionally mature cell types, we cultured the cells under cardiomyocyte‐like cell‐differentiating conditions. After 14 days of culture, we confirmed the presence of clusters of beating cells (Supplementary data).

## Discussion

Male infertility is regarded as a major public health concern because it affects 15 % of couples worldwide. More than half of all infertility cases are associated with male factor infertility [10]. In addition, approximately 15 % of idiopathic male factor infertility is due to azoospermia [11]. Patients with KS [1, 12, 13, 14] present with low serum testosterone, high luteinizing hormone (LH) and follicle‐stimulating hormone (FSH) levels, and often elevated estradiol levels. Almost all KS patients exhibit azoospermia, and few sperm cells are isolated when these patients undergo micro‐TESE. In addition, KS patients have an increased risk of developing diseases such as diabetes, cardiovascular disease, and cancer, and there is an increased risk for chromosomal abnormalities in their offspring [1].

A better understanding of the mechanism(s) underlying KS will facilitate the development of novel treatment options for patients with this condition. Here we developed KS‐derived iPS cells, which could be useful for mechanistic studies of KS. We previously generated iPS cells derived from adult human testicular tissue using lentiviral vectors expressing OCT4, SOX2, KLF4, and C‐MYC [15]. We also succeeded in generating iPS cells derived from the testicular tissue of a KS patient using the same system [6]. In addition, another group generated iPS cells derived from a KS patient using retroviral vectors [16]. However, several characteristics of *Lentivirus* limit its use for generating iPS cells. For example, *Lentivirus* infects dividing as well as non‐dividing cells, and integrates into the host genome, resulting in strong transgene expression. In addition, *Lentiviral* gene expression is resistant to silencing. Thus, the genomic integration of *C*‐*MYC* has the potential to increase the tumorigenicity of the iPS cells. To generate higher quality iPS cells, here we used the Sendai virus (SeV) system. SeV is a negative sense, single‐stranded RNA virus, which replicates in the cytoplasm without integrating into the host genome, and induces iPS cells efficiently.

In addition to reducing the potential tumorigenicity of the iPS cells, various issues regarding the use of iPS cells still need to be addressed. Recent studies have shown that human somatic cells and iPS cells accumulate both epigenetic modifications and genetic mutations that affect their properties and could impact their research utility and clinical safety [17, 18, 19], and one study showed that iPS cell lines exhibit epigenetic variations and broad pluripotency [20]. Thus, extensive genetic screening and characterization of the iPS cells is required before using them in research or clinical studies.

Thus, among our evaluations of the KS patient‐derived iPS cells, we tested their ability to undergo cardiomyocyte‐like cell differentiation in vitro. Our finding that these cells could differentiate into cardiomyocyte‐like cells was a strong indication of their potential for pluripotency. In addition, we showed that these cells were capable of undergoing embryoid formation in vitro and of forming tumors in vivo, containing the three germ layers. Taken together, these findings suggested that our KS‐derived iPS cells were high‐quality pluripotent iPS cells.

Many researchers previously reported that both human ES cells and iPS cells can undergo germ cell differentiation in vitro [21, 22, 23, 24, 25, 26]. It was also recently reported that the transplantation of normal and azoospermic factor (AZF)‐deficient iPS cells into the murine seminiferous tubule results in the formation of germ‐cell‐like cells, whereas iPS cells outside the tubule fail to differentiate into germ‐cell‐like cells [27]. In addition, the AZF‐deficient iPS cells form fewer primordial germ‐cell‐like cells, which exhibit defective gene expression. Thus, the murine seminiferous tubules can direct the formation and maintenance of germ‐cell‐like cells from human iPS cells.

Future studies, such as those evaluating the ability of KS patient‐derived iPS cells to undergo germ cell differentiation when transplanted into mice, will be required to further characterize these cells for their utility in mechanistic studies and potential therapeutic use.

## Acknowledgments

This study was supported in part by a Grant‐in‐Aid for Young Scientists (B) of the Japan Society for the Promotion of Science (JSPS) and a grant from the Strategic Research Foundation Grant‐aided Project for Private Schools at Heisei 23rd from the Ministry of Education, Culture, Sports, Science and Technology of Japan, 2011–2015. H.K. supervised the entire project.

## Compliance with ethical standards

### Conflict of interest

Toshihiro Shimizu, Mami Shiohara, Toshihiro Tai, Koichi Nagao, Koichi Nakajima, and Hideyuki Kobayashi declare that they have no conflict of interest.

### Human rights statements and informed consent

All procedures followed were in accordance with the ethical standards of the responsible committee on human experimentation (institutional and national) and with the Helsinki Declaration of 1964 and its later amendments. Informed consent was obtained from all patients included in the study.

### Animal studies

All institutional and national guidelines for the care and use of laboratory animals were followed.

## Supporting information

Supplementary data: Demonstration of beating cardiomyocyte‐like cells derived from the KS iPS cells (MP4 8195 kb)
